# Probiotics and Prebiotics in Restoring Gastrointestinal Microbiota Dysbiosis: Emerging Alternatives to Antibiotic Therapy

**DOI:** 10.1155/ijfo/6956918

**Published:** 2026-11-14

**Authors:** Tigistu Goa, Tesfaye Wolde, Elshaday Sahilu

**Affiliations:** ^1^ Department of Biology College of Natural and Computational Science Wolkite University Wolkite Ethiopia wku.edu.et; ^2^ School of Life Science Tsinghua Shenzhen International Graduate School Shenzhen China; ^3^ Department of Biotechnology College of Natural and Computational Science Wolkite University Wolkite Ethiopia wku.edu.et

**Keywords:** dysbiosis, gut microbiota, gut–organ axis, personalized medicine, prebiotics, probiotics, short-chain fatty acids

## Abstract

The human gastrointestinal (GI) microbiota is a complex and dynamic microbial ecosystem that plays a fundamental role in maintaining host metabolism, immune homeostasis, intestinal barrier integrity, and protection against pathogenic microorganisms. Disruption of this microbial community, known as gut dysbiosis, has been implicated in the pathogenesis of numerous GI and systemic diseases, including inflammatory bowel disease (IBD), irritable bowel syndrome (IBS), *Clostridioides difficile* infection, obesity, type 2 diabetes mellitus, colorectal cancer, and nonalcoholic fatty liver disease. Although antibiotics remain indispensable for the treatment of bacterial infections, their indiscriminate use frequently disrupts gut microbial composition, reduces microbial diversity, promotes opportunistic pathogen overgrowth, and accelerates the emergence of antimicrobial resistance. These limitations have stimulated increasing interest in microbiota‐targeted interventions as safer and more sustainable therapeutic strategies. Probiotics and prebiotics have emerged as promising approaches for restoring gut microbial homeostasis through complementary mechanisms. Probiotics inhibit pathogen colonization, enhance intestinal barrier function, produce antimicrobial metabolites, and modulate host immune responses. Prebiotics selectively stimulate the growth and metabolic activity of beneficial microorganisms, promoting the production of short‐chain fatty acids (SCFAs) that support intestinal and systemic health. Increasing evidence from experimental studies, randomized clinical trials, and meta‐analyses demonstrates that these microbiota‐directed interventions can reduce antibiotic‐associated diarrhea, improve symptoms of IBD and IBS, enhance *Helicobacter pylori* eradication, decrease recurrence of *C. difficile* infection, and contribute to the management of several metabolic disorders. Despite these promising findings, important challenges remain regarding strain specificity, optimal dosage, treatment duration, product standardization, regulatory oversight, and interindividual variability in treatment response. Emerging advances in metagenomics, metabolomics, artificial intelligence, and precision medicine are facilitating the development of personalized microbiome‐based therapies that may further enhance clinical outcomes. This review critically summarizes the current understanding of GI microbiota dysbiosis, discusses the mechanisms through which probiotics and prebiotics restore microbial balance, evaluates their clinical applications as alternatives or adjuncts to antibiotic therapy, and highlights existing challenges and future research directions for microbiome‐based therapeutics.

## 1. Introduction

The human gastrointestinal tract (GIT) harbors a complex and dynamic community of microorganisms collectively known as the gut microbiota [1]. The gut microbiota produces short‐chain fatty acids (SCFAs), synthesizes essential vitamins, modulates immune responses, and produces antimicrobial peptides (AMPs) [2]. They also convert primary bile acids into secondary bile acids that regulate lipid digestion, cholesterol metabolism, and metabolic homeostasis [3, 4].

Emerging evidence suggested that the gut contributes to the regulation of homeostasis beyond the local intestinal environment, influencing distant organs including the liver, lungs, central nervous system (CNS), and skeletal muscle [5]. Serotonin is crucial for brain and GIT function. Inside GIT, enterochromaffin cell is responsible for storing 90% of serotonin, and its amount is highly affected by intestinal microbiota [6]. Although substantial evidence supports the existence of gut–organ communication networks, the precise molecular mechanisms governing these interactions remain under active investigation [7].

A stable and balanced microbial community is essential for maintaining gastrointestinal homeostasis. However, this balance can be disrupted by various factors such as poor diet, infections, stress, aging, and particularly the use of antibiotics [8, 9]. This disruption, known as gut microbiota dysbiosis, has been associated with a wide range of diseases including inflammatory bowel diseases (IBD), Crohn′s disease (CD), Alzheimer′s disease (AD), Parkinson′s disease (PD), nonalcoholic fatty liver disease, allergies, and gastric cancer [10, 11]. Research finding showed that severity of COVID‐19 infection is associated with the increment of opportunistic pathogens and reduction of beneficial microbes. It is hypothesized that gut microbiota disturbance is one of the risk factor for the severity of COVID‐19 infection [12, 13]. Solid research evidence confirmed dysbiosis of gut microbiota (*Lactobacillus* and *Bifidobacterium*) in COVID‐19 patients [14].

Antibiotics have long been the primary therapeutic approach for managing bacterial infections [15]. Although highly effective in eliminating pathogenic organisms, antibiotics are nonselective in their action and often eradicate beneficial gut microbes alongside harmful ones [16]. This broad‐spectrum activity can lead to reduced microbial diversity, altered metabolic functions, and increased vulnerability to opportunistic infections such as *Clostridium difficile* (*C. difficile*) [17] (Figure 1).

**Figure 1:**
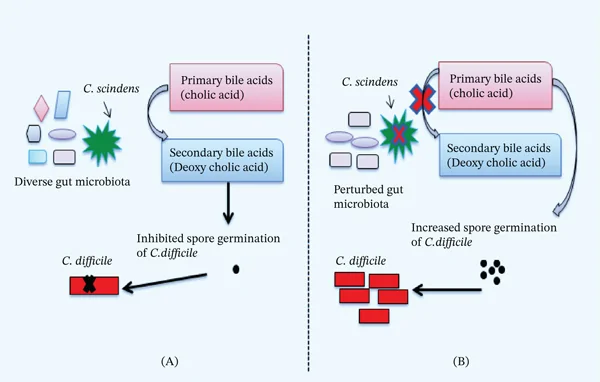
(A) Eubiosis (balanced gut microbiota) and (B) dysbiosis (disturbed gut microbiota). Created by the authors using Microsoft Paint and Microsoft Word (Microsoft Corporation, Redmond, Washington, United States). (A) Eubiosis: A diverse and relatively balanced gut microbiota includes bile‐acid–transforming bacteria such as *Clostridium scindens* (*C. scindens*), which converts primary bile acids, including cholic acid, into secondary bile acids, including deoxycholic acid. Secondary bile acids inhibit the germination of *C. difficile* spores, thereby contributing to colonization resistance and limiting *C. difficile* expansion. (B): Dysbiosis following broad‐spectrum antibiotic exposure: Antibiotic‐induced disruption of the gut microbiota can reduce or eliminate bile‐acid–transforming bacteria such as *C. scindens*, resulting in reduced conversion of primary to secondary bile acids.

The resulting alteration in the bile acid pool is associated with increased germination of *C. difficile* spores and facilitates subsequent *C. difficile* proliferation [18]. Antibiotic‐induced gut dysbiosis may deplete beneficial commensal bacteria and disrupt microbiota–host interactions that regulate AMP production, thereby weakening mucosal defense against pathogens [19]. Moreover, repeated or inappropriate antibiotic use contributes to the growing global concern of antimicrobial resistance (AMR), further limiting treatment options for infectious diseases. These challenges have intensified interest in alternative strategies that can restore or maintain microbial balance without the detrimental effects associated with antibiotics [20–22].

In this context, probiotics and prebiotics are increasingly being studied for their potential to modulate the gut microbiota and improve health outcomes [23]. Probiotics are live microorganisms that, when administered in adequate amounts, confer health benefits [24]. Common probiotic strains include species of *Lactobacillus, Bifidobacterium*, and *Saccharomyces* [25]. These organisms exert beneficial effects through multiple mechanisms, such as competitive exclusion of pathogens, enhancement of intestinal barrier function, production of antimicrobial substances, and modulation of host immune responses [26].

Prebiotics are nondigestible food components, typically dietary fibers such as inulin, fructooligosaccharides (FOS), and galactooligosaccharides (GOS), that selectively stimulate the growth and activity of beneficial gut bacteria [27, 28]. By serving as substrates for fermentation, prebiotics promote the production of SCFAs like butyrate, acetate, and propionate, which play critical roles in maintaining colonic health, regulating inflammation, and supporting metabolic processes [29, 30].

Despite the fact that existing literature reviews predominantly provide descriptive overviews of microbiota modulating interventions, there is limited emphasis on conflicting evidence, mechanistic uncertainties, and translational challenges. Moreover, few studies address the variability in host response or the limitations of current clinical data [31–33]. In addition, assessing the effectiveness of probiotics in a specific disease is difficult due to heterogeneity of different studies and clinical trials about prebiotics in treating GIT‐related diseases are not sufficiently conducted [34]. This review is aimed at critically evaluating the mechanisms through which probiotics and prebiotics restore gut microbial balance, assessing their therapeutic applications, and identifying translational challenges and factors contributing to interindividual variability in treatment responses.

## 2. Probiotics in Reshifting Dysbiosis: Mechanisms of Action of Probiotics

The mechanisms discussed in this section are supported by evidence of different types and strengths, ranging from in vitro experiments and animal models to translational and, in some cases, human studies. Mechanistic findings demonstrate biological plausibility but do not necessarily establish clinical efficacy. Accordingly, the evidence presented below is interpreted with attention to experimental context, strain specificity, host characteristics, and the extent to which findings have been reproduced or validated in human studies. Where clinical evidence is available, it is distinguished explicitly from preclinical or mechanistic observations

### 2.1. Restoration of Beneficial Bacterial Populations

Probiotics enhance microbial diversity and community richness; a hallmark of a resilient and healthy microbiota. These interventions maintain a balanced ecosystem, prevent opportunistic pathogen growth, and support gut stability. It promotes the growth of beneficial species such as *Lactobacillus*, *Bifidobacterium*, and other commensals [35, 36].

Although the concept that probiotics may promote microbiota recovery is biologically plausible, recent evidence indicates that restoration of microbial diversity is neither consistent across populations nor necessarily clinically meaningful. A recent systematic review and meta‐analysis of randomized controlled trials found no significant overall effect of probiotic supplementation on Shannon, Chao1, observed operational taxonomic units, or Simpson diversity indices in healthy populations, highlighting the variability of responses across probiotic interventions and study populations [37]. However, evidence for restoration of specific beneficial bacterial populations remains heterogeneous. A systematic review and meta‐analysis of 29 randomized controlled trials involving 1633 older adults found that probiotic supplementation significantly increased *Bifidobacterium* abundance (SMD: 0.40; 95% CI: 0.06–0.75; *p* < 0.05), whereas also increasing Shannon diversity, suggesting that microbiota responses may depend on population and intervention characteristics [36].

Moreover, probiotic‐associated alterations in the gut microbiota may not necessarily represent durable ecological restoration. A longitudinal intervention study of short‐term probiotic supplementation demonstrated dynamic changes in microbial diversity and community characteristics during supplementation and subsequent follow‐up, highlighting the temporal variability of microbiota responses to probiotic exposure [38].

Importantly, increased *α*‐diversity should not be interpreted as a direct proxy for microbiota health or functional recovery. The complexity and dynamic nature of the microbiome indicate that health‐related interpretation requires consideration of community composition, functional capacity, ecological interactions, and host‐related outcomes rather than diversity alone [39, 40]. Thus, future studies should evaluate probiotic efficacy using integrated measures of taxonomic composition, microbial function, ecological resilience, and clinically relevant outcomes rather than relying solely on diversity indices.

### 2.2. Enhancement of Intestinal Barrier Function and Epithelial Integrity

The intestinal barrier is a multilayered system consisting of the mucus layer, intestinal epithelial cells (IECs), intercellular junctions, and underlying immune components. Goblet cells produce mucus, which forms a protective interface over the epithelium, whereas Paneth cells secrete AMPs, including defensins and lysozyme. The lamina propria contains antigen‐presenting cells, phagocytic cells, and B and T lymphocytes that contribute to mucosal immune surveillance [41, 42]. Together, these components limit the adherence and translocation of pathogens and microbial products while maintaining controlled interactions between the intestinal microbiota and host tissues.

Experimental evidence indicates that selected probiotic strains can modulate intestinal epithelial‐cell proliferation and apoptosis, thereby contributing to epithelial renewal and maintenance of the mechanical barrier [43]. Probiotics can also modulate tight‐junction proteins, including occludin, claudins, and zonula occludens‐1 (ZO‐1), thereby influencing paracellular permeability and potentially limiting the translocation of luminal pathogens, toxins, and antigens across the intestinal epithelium [44].

In addition, certain probiotic strains can interact with the intestinal mucus barrier and mucin‐associated structures. For example, specific *Bifidobacterium bifidum* strains have been shown to interact with the O‐glycosylated mucin MUC13, adhere to and penetrate the mucus layer, and enhance intestinal epithelial barrier integrity, highlighting the strain‐specific nature of probiotic–mucus interactions [45]. Some probiotic strains may also stimulate epithelial antimicrobial responses, including the production of AMPs such as defensins. For example, *Bacillus subtilis* has been shown to induce human *β*‐defensin‐2 expression in IECs [46]. In addition, adhesion of certain probiotic bacteria, particularly *Bifidobacterium* species, to the mucus layer may contribute to pathogen exclusion [47]. Persistent disruption of intestinal barrier integrity has been associated with GI diseases, including IBD and celiac disease, as well as several systemic inflammatory and metabolic disorders. However, altered intestinal permeability does not by itself establish a causal role in disease development or demonstrate that restoration of barrier function will necessarily improve clinical outcomes [48, 49].

Among the proposed probiotic mechanisms, enhancement of intestinal barrier function has a relatively substantial evidence base, although the magnitude and consistency of effects vary according to probiotic strain, formulation, dose, intervention duration, host characteristics, and outcome measure. Experimental and clinical evidence indicates that probiotics can influence epithelial barrier integrity through modulation of tight‐junction proteins, epithelial signaling, mucus interactions, and inflammatory pathways [44]. However, evidence from clinical studies is not uniform. A systematic review of randomized controlled trials and animal studies focusing on overweight and obesity reported inconsistent effects of probiotic supplementation on intestinal permeability, with improvements observed for some outcomes but not consistently across studies [50]. Similarly, a systematic review and meta‐analysis of randomized controlled trials found significant improvements in selected barrier‐related outcomes, including transepithelial electrical resistance and serum zonulin, but emphasized the need for further high‐quality trials before definitive conclusions can be drawn [51].

Importantly, these effects cannot be generalized across probiotic genera or strains. Specific strains of *Lactobacillus*, *Bifidobacterium*, and other probiotic microorganisms have demonstrated barrier‐modulating activity, but the magnitude and mechanisms of these effects vary among strains and experimental contexts [44, 52]. Thus, an improvement in an individual permeability‐related biomarker should not automatically be interpreted as evidence of generalized restoration of intestinal barrier function. A further limitation concerns the translational significance of these physiological changes. Although altered intestinal permeability is associated with several gastrointestinal and metabolic disorders, the clinical significance of changes in permeability‐related biomarkers remains incompletely established [49]. Moreover, mechanistic evidence derived from in vitro and animal models has limited direct clinical applicability because these models do not fully reproduce the complex human intestinal environment and its interactions among epithelial cells, mucus, immune components, and the resident microbiota [44].

Collectively, current evidence supports probiotic‐mediated intestinal barrier enhancement as a biologically plausible and potentially clinically relevant mechanism, but not as a universal effect across probiotic strains or patient populations. Well‐designed, adequately powered clinical trials using validated measures of intestinal permeability together with clinically meaningful outcomes are therefore needed to determine which probiotic strains, doses, and patient populations are most likely to derive clinically meaningful benefits [44, 51].

### 2.3. Competitive Exclusion of Pathogenic Bacteria

Competitive exclusion is an important mechanism by which probiotics may inhibit pathogen colonization through competition for adhesion sites and nutrients. Probiotic adhesion to intestinal epithelial surfaces can occupy host‐binding sites and thereby interfere with the attachment and colonization of pathogenic bacteria [53].

Certain probiotic strains express adhesion‐associated surface proteins, including mucus‐binding proteins (MUB) and mucus adhesion‐promoting protein (MapA), which facilitate interactions with intestinal mucus and mucins. Other surface‐associated proteins, including enolase and glyceraldehyde‐3‐phosphate dehydrogenase (GAPDH), have also been implicated in adhesion to intestinal epithelial or extracellular‐matrix components [54, 55]. Such adhesion may contribute to competitive exclusion by allowing probiotic strains to occupy or interact with host binding sites and potentially limit pathogen attachment [54].

Probiotic lactic acid bacteria (pLAB) can produce antimicrobial compounds including organic acids, carbon dioxide (CO_2_), bacteriocins, hydrogen peroxide, and diacetyl. The main inhibitory effect of pLAB is attributed to the production of lactic acid and the consequent reduction of luminal pH [56, 57]. Among bacteriocins, nisin and pediocin are FDA‐approved as natural food preservatives due to their antimicrobial activity against gram‐positive bacteria, particularly foodborne pathogens such as *Listeria monocytogenes*, *Clostridium* spp., and *Staphylococcus aureus* [58]. However, these bacteriocins are generally ineffective against gram‐negative bacteria because the outer membrane is impermeable to most bacteriocins, and their spectrum is typically limited to closely related species [59]. Furthermore, bacteriocin efficacy varies considerably between strains and environmental conditions, and most evidence is derived from laboratory studies, with limited confirmation in complex human gut ecosystems [60–62].

Overall, probiotic‐mediated pathogen exclusion involves multiple complementary mechanisms, but their relative contribution and translational relevance vary substantially according to strain characteristics, host factors, pathogen susceptibility, dose, and gastrointestinal environmental conditions. The major mechanisms and their supporting evidence, consistency, and translational relevance are illustrated in Figure 2 and summarized in Table 1.

**Figure 2:**
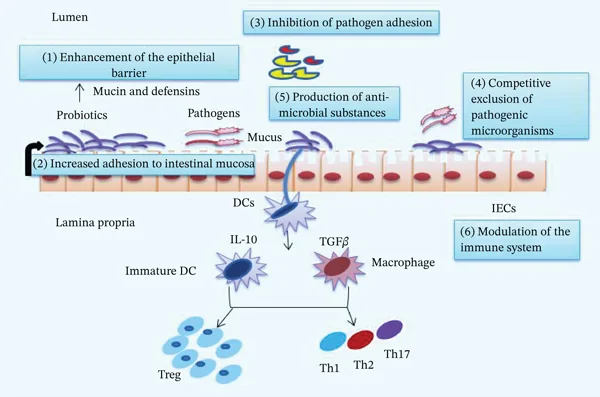
Mechanisms of probiotic action in the gastrointestinal tract. Schematic representation of the major mechanisms through which probiotics may influence intestinal health, including (1) enhancement of the intestinal epithelial barrier through promotion of mucin and antimicrobial peptide production; (2) adhesion to the intestinal mucosa, potentially limiting pathogen attachment; (3) inhibition of pathogen adhesion to intestinal epithelial cells; (4) competitive exclusion of pathogens through competition for adhesion sites and nutrients; (5) production of antimicrobial compounds, including organic acids and other inhibitory metabolites; and (6) modulation of host immune responses through interactions with intestinal epithelial and immune cells. The illustration also depicts immune‐related interactions involving dendritic cells (DCs), macrophages, regulatory T cells (Tregs), and T‐helper cell subsets (Th1, Th2, and Th17), as well as the involvement of cytokines such as interleukin‐10 (IL‐10) and transforming growth factor‐beta (TGF‐*β*). Adapted/redrawn from Bermudez‐Brito et al. [26], Annals of Nutrition and Metabolism, 61(2), 160–174, 10.1159/000342079. This figure/illustration content has been modified from the original. Copyright: 2012 Karger Publishers, Basel, Switzerland. Reused with permission from S. Karger AG. The final published version of the original article is available at https://karger.com/?doi=10.1159/000342079.

**Table 1:** Submechanisms of probiotic‐mediated pathogen exclusion: Evidence levels, consistency, translational gaps, and clinical relevance.

Submechanism	Evidence level for mechanism	Consistency	Translational gap	Clinical relevance	References
Competition for adhesion sites	Level II–III*	Moderate	Adhesion is highly strain‐specific and depends on mucus composition, receptor availability, intestinal location, and resident microbiota. In vitro adhesion does not necessarily predict sustained colonization or pathogen exclusion in humans.	Potentially relevant to prevention of pathogen colonization, but clinical importance remains strain‐ and pathogen‐dependent.	[54]
Competition for nutrients	Level III*	Low–moderate	Nutrient availability and utilization differ substantially between laboratory models and the intestinal ecosystem. Although nutrient competition contributes to microbiota‐mediated colonization resistance, the extent to which administered probiotic strains independently reproduce this effect in vivo remains uncertain.	May contribute to colonization resistance, but direct probiotic‐specific clinical evidence remains limited.	[63]
Lactic acid production	Level II–III*	Moderate	Antimicrobial activity depends on local concentration, pH, buffering capacity, pathogen susceptibility, and the ability of the producing strain to generate sufficient acid in vivo.	May contribute to pathogen suppression under favorable local conditions, but its independent contribution to clinical outcomes is difficult to establish.	[64, 65]
Bacteriocin production	Level II–III*	Low–moderate	Bacteriocins differ substantially in spectrum, stability, expression, and activity. Degradation, dilution, and interactions with resident microbiota may limit activity in the gastrointestinal environment.	Potentially important for selected bacteriocin‐producing strains, but broad clinical generalizability remains uncertain.	[66]
Hydrogen peroxide production (H_2_O_2_)	Level II–III*	Low–moderate	Evidence from experimental models indicates that probiotic‐derived H_2_O_2_ may contribute to antimicrobial activity, but its effects depend on strain‐specific production, local oxygen availability, concentration, antioxidant activity, and interactions with catalase‐producing microorganisms.	May contribute to microbial control under specific physiological conditions, but its independent contribution to clinical benefit remains unestablished.	[67, 68]

*Note:* Evidence level for mechanism: Evidence levels were assigned according to the highest level of direct mechanistic evidence identified in the underlying literature cited or reviewed by the referenced sources. Level I indicates direct mechanistic evidence from human clinical or intervention studies; Level II indicates direct evidence from animal or other in vivo experimental studies; and Level III indicates evidence derived primarily from in vitro, cell‐based, biochemical, or other experimental models. Combined designations (e.g., Level II–III) indicate that the specific mechanism has been investigated in both animal/in vivo and in vitro experimental systems, whereas direct human mechanistic evidence is lacking. Asterisk “*” denotes evidence‐level classifications refer to the underlying mechanistic evidence and do not imply that the cited review itself constitutes evidence at that level. Consistency: Consistency reflects the reproducibility of findings across studies and experimental conditions. High = generally concordant findings; moderate = generally supportive but with important strain‐, dose‐, pathogen‐, host‐, or model‐dependent variation; low = limited, inconsistent, or highly context‐dependent evidence. Translational gap: Refers to limitations in extrapolating mechanistic findings from experimental models to the complex human gastrointestinal environment and clinical settings. Clinical relevance: Refers to the potential contribution of each mechanism to pathogen suppression or host protection in humans, rather than evidence that the mechanism independently produces a clinical benefit.

The major antimicrobial compounds produced by pLAB and their proposed mechanisms of action against pathogenic bacteria are summarized in Table 2.

**Table 2:** Antimicrobial compounds produced by lactic acid bacteria and their proposed molecular mechanisms of action against pathogenic bacteria.

Representative LAB producer(s)	Antimicrobial compound	Proposed molecular mechanism of action against pathogenic bacteria	References
Heterofermentative *Leuconostoc* spp. and other LAB	Acetic acid	Undissociated acetic acid can permeate the bacterial cytoplasmic membrane and dissociate intracellularly, contributing to cytoplasmic acidification, disruption of proton homeostasis, and impairment of cellular metabolism.	[56, 69]
*Lactobacillus*, *Lactiplantibacillus*, *Leuconostoc*, and other lactic‐acid–producing LAB	Lactic acid	Undissociated lactic acid can permeate bacterial membranes and dissociate intracellularly, altering intracellular proton balance and contributing to cytoplasmic acidification and metabolic inhibition.	[69, 70]
Heterofermentative LAB	Carbon dioxide (CO_2_)	CO_2_ can inhibit susceptible bacteria by creating a locally anaerobic environment, interfering with cellular enzyme activity, and contributing to disruption of bacterial membrane function.	[71]
H_2_O_2_‐producing LAB	Hydrogen peroxide (H_2_O_2_)	H_2_O_2_ exerts oxidative stress on susceptible bacteria, resulting in oxidative damage to essential cellular components and impairment of bacterial viability.	[71, 72]
Bacteriocin‐producing LAB	Bacteriocins	Bacteriocins interact with the bacterial membrane, leading to pore formation and membrane permeabilization, which causes leakage of intracellular ions and ATP, ultimately resulting in loss of membrane integrity and bacterial viability.	[73]
Diacetyl‐producing LAB	Diacetyl(2,3‐butanedione)	Diacetyl reacts with arginine residues in target proteins, potentially compromising protein structure and function and interfering with arginine‐dependent cellular metabolism, thereby inhibiting bacterial growth.	[74]

### 2.4. Immunomodulatory Mechanisms

Probiotics can interact with the host immune system through microbe‐associated molecular patterns (MAMPs), lipopolysaccharides (LPS), peptidoglycans and flagellin recognition by pattern recognition receptors (PRRs), including Toll‐like receptors (TLRs) and NOD‐like receptors (NLRs) on intestinal epithelial and immune cells. Activation initiates intracellular signaling cascades involving MyD88 and downstream pathways (NF‐*κ*B and MAPKs), regulating cytokine production and immune responses [75, 76].

Probiotics modulate both innate and adaptive immunity by enhancing anti‐inflammatory responses and suppressing excessive inflammation. This includes increased production of regulatory cytokines (IL‐10 and TGF‐*β*), modulation of dendritic cell (DC) function, regulatory T cell (Treg) activity, and immunoglobulin production [77, 78]. Despite these promising findings, clinical evidence remains heterogeneous and the immunomodulatory effects of probiotics are not consistently observed across different populations or disease conditions. This variability underscores the importance of host‐specific factors and highlights the need for personalized approaches [79].

The immunomodulatory effects of probiotics are among the most extensively studied but also the most heterogeneous. An umbrella meta‐analysis of randomized controlled trials found that probiotic supplementation significantly reduced TNF‐*α* (ES = −0.35; 95% CI: −0.50 to −0.20; *p* < 0.001), IL‐6 (ES = −0.36; 95% CI: −0.59 to −0.13; *p* = 0.002), and C‐reactive protein (CRP) (ES = −1.02; 95% CI: −1.23 to −0.80; *p* < 0.001); however, substantial heterogeneity was observed for TNF‐*α* (*I* ^2^ = 75.6*%*), IL‐6 (*I* ^2^ = 85.6*%*), and CRP (*I* ^2^ = 94.1*%*), indicating considerable variability across studies [80]. Similarly, a systematic review and meta‐analysis of 26 RCTs (*n* = 1891) reported reductions in CRP, TNF‐*α*, and IL‐6 following probiotic supplementation, while noting persistent heterogeneity and limitations related to differences in probiotic types and study populations [51]. Collectively, these findings support a potential anti‐inflammatory effect of probiotics but indicate that the magnitude and consistency of immunomodulation vary substantially according to strain, host characteristics, clinical condition, and intervention design [51, 80].

Strain‐specific effects are also evident. *Lacticaseibacillus rhamnosus* GG and *Bifidobacterium longum* subsp. *infantis* 35624 have been associated with regulatory immune responses, including modulation of Treg activity and IL‐10 production in selected experimental and clinical settings. However, these effects are not consistently observed across probiotic strains, emphasizing that immunomodulatory effects should not be generalized across species or commercial products [81–83]. A recent commentary by Cano and García Menendez [79] highlighted the challenges of translating microbiome‐targeted interventions into clinically meaningful health claims, emphasizing the importance of appropriate clinical endpoints, objective biomarkers, and careful consideration of biological and methodological variability. These limitations underscore the need for better patient stratification, mechanistic biomarkers, and personalized approaches when evaluating probiotic interventions [79].

According to the work of Harzallah et al. [84] and Hardy et al. [85], the intestinal immune system continuously monitors luminal contents to maintain homeostasis and protect the host from potential pathogens. Antigen sampling occurs through several complementary mechanisms. First, DCs located in the lamina propria can extend transepithelial dendrites between epithelial cells into the intestinal lumen, enabling direct sampling of microbial antigens and other luminal substances. Second, IECs can internalize small quantities of luminal material through pinocytosis and other endocytic processes, facilitating antigen delivery to underlying immune cells. Third, specialized microfold (M) cells within the follicle‐associated epithelium of Peyer′s patches selectively transport luminal antigens, microorganisms and particulate matter to antigen‐presenting cells, thereby initiating mucosal immune responses. Recent studies have highlighted the critical role of M cells and DC‐mediated antigen sampling in maintaining intestinal immune homeostasis, promoting tolerance to commensal microbiota, and generating protective immune responses against enteric pathogens [85–87] (Figure 3).

**Figure 3:**
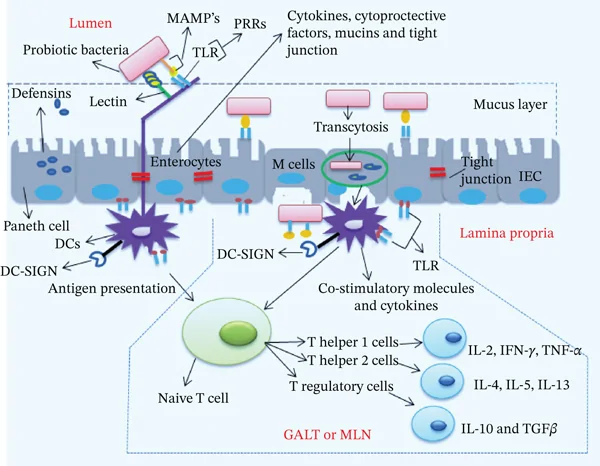
Mechanisms of probiotic‐mediated immunomodulation at the intestinal mucosa. Created by the authors using Microsoft Paint and Microsoft Word (Microsoft Corporation, Redmond, Washington, United States). The illustration depicts probiotic bacteria and their molecular components within the intestinal lumen and their interactions with the mucus layer, intestinal epithelial cells (IECs), Paneth cells, dendritic cells (DCs), and T cells in the lamina propria and gut‐associated lymphoid tissue (GALT) or mesenteric lymph nodes (MLN). The figure illustrates epithelial recognition and signaling, antimicrobial peptide release, luminal antigen sampling through IECs and microfold (M) cells, dendritic‐cell antigen presentation, and differentiation of naive T cells into T helper 1 (Th1), T helper 2 (Th2), and regulatory T (Treg) cell subsets. Abbreviations: AMPs, antimicrobial peptides; DCs, dendritic cells; DC‐SIGN, dendritic cell‐specific intercellular adhesion molecule‐3‐grabbing nonintegrin; GALT, gut‐associated lymphoid tissue; IECs, intestinal epithelial cells; IFN‐*γ*, interferon‐gamma; IL, interleukin; MAMPs, microbe‐associated molecular patterns; MLN, mesenteric lymph nodes; PRRs, pattern recognition receptors; TGF‐*β*, transforming growth factor‐beta; Th1, T helper 1 cell; Th2, T helper 2 cell; TLR, Toll‐like receptor; TNF‐*α*, tumor necrosis factor‐alpha; Treg, regulatory T cell. Figure elements: Arrows indicate signaling, transport, or differentiation; dashed lines delineate anatomical compartments; and red bars represent epithelial barrier structures, including tight‐junction complexes between IECs.

### 2.5. Metabolic Pathways and Microbial Metabolites

Gut microorganisms metabolize dietary fibers and resistant starches to produce SCFAs primarily acetate, propionate, and butyrate. Those metabolites regulate luminal pH, act as signaling molecules, influencing immune responses and host energy metabolism. SCFAs maintain intestinal barrier integrity and systemic metabolic homeostasis [4, 88].

Bile‐acid transformation is facilitated by gut microbiota. Primary bile acids synthesized in the liver are converted into secondary bile acids through microbial deconjugation and dehydroxylation. Modified bile acids regulate lipid metabolism and immune signaling via receptors such as farnesoid X receptor (FXR) and Takeda G protein‐coupled receptor 5 (TGR5) [89, 90]. Gut microbes contribute to host nutrition through biosynthesis of essential B‐group vitamins and vitamin K supporting enzymatic reactions, energy metabolism, and neurological function [91, 92].

They also regulate tryptophan metabolism, converting it into bioactive compounds such as indoles and derivatives, which activate host receptors including the aryl hydrocarbon receptor (AhR), influencing immune responses, epithelial barrier integrity, and systemic processes [93, 94].

Microbial communities are highly interdependent, relying on metabolic cross‐feeding. Metabolites produced by breakdown of complex carbohydrates serve as substrates for other species. Primary carbohydrate degraders generate SCFAs utilized by secondary fermenters, creating a functional network that enhances ecosystem resilience [95, 96].

SCFA production is an important and frequently investigated consequence of prebiotic fermentation; however, the magnitude and consistency of the response vary according to substrate characteristics, dose, and host microbiota. Recent systematic evidence indicates that specific dietary fibers can increase individual SCFAs, but these effects are not uniform across interventions, with responses influenced by fiber structure, intrinsic properties, and dosage [97]. Importantly, increased production of microbial metabolites does not necessarily translate into clinical benefit. Although butyrate has well‐established anti‐inflammatory and epithelial effects, clinical evidence for butyrate supplementation in ulcerative colitis remains limited. A recent double‐blind randomized controlled trial in patients with active ulcerative colitis reported significant reductions in disease severity and inflammatory markers following sodium‐butyrate supplementation compared with placebo; however, the relatively small sample size (*n* = 36) highlights the need for larger and longer term trials to establish its clinical efficacy [98]. Similarly, bile‐acid transformation and microbial tryptophan metabolism are increasingly recognized as important host‐microbiota signaling pathways, but their clinical implications remain incompletely defined [99]. Recent experimental work further demonstrates that tryptophan‐derived indoles can participate in microbial cross‐feeding, illustrating the complexity of metabolite‐mediated interactions within the gut ecosystem [100]. Collectively, these findings indicate that changes in SCFAs and other microbial metabolites should be interpreted primarily as mechanistic evidence rather than as direct evidence of clinical efficacy.

### 2.6. Gut–Organ Axis Communication

The gut–brain axis involves the production of neuroactive compounds, such as serotonin and gamma‐aminobutyric acid, along with vagal nerve signaling, enabling bidirectional communication that influences brain function, behavior, and stress responses. In vivo study confirmed that *Bifidobacterium dentium* was used to increase the serotonergic levels in mice. The results showed increased fecal acetate, which regulates key components of serotonergic system [101].

The gut–liver axis operates when microbial metabolites and components are transported via the portal circulation to the liver, where they regulate metabolic processes, immune activity, and bile acid signaling. The gut–lung axis operates via immune cross‐talk, whereby gut‐derived mediators influence respiratory immunity and susceptibility to infections and inflammatory conditions. Those pathways highlight the microbiota′s role as a coordinator of systemic homeostasis with SCFA, secondary bile acids, and tryptophan derivatives—acting as key signaling molecules that exert regulatory effects on distant organs [102–104].

The gut–organ axis concept is biologically plausible and supported by substantial experimental and emerging clinical evidence; however, translation into consistent human clinical outcomes remains incomplete. Much of the available human evidence is associative, and establishing causality remains challenging because microbiome composition, host characteristics, diet, medication use, and disease status can influence both microbiota profiles and clinical outcomes [105, 106].

In the gut–brain axis, probiotics have been reported to influence neuroactive metabolites, neurotransmitter signaling, immune pathways, and brain activity, but clinical findings remain heterogeneous. Systematic evidence from randomized trials suggests that probiotic interventions may influence brain‐related outcomes, although interpretation is constrained by small sample sizes, heterogeneous probiotic formulations, and methodological variability [107]. Similarly, although probiotics may influence serotonin‐related pathways, current evidence does not establish that probiotic‐induced changes in serotonergic signaling consistently translate into clinically meaningful neurological or gastrointestinal outcomes in humans [6]. Importantly, changes in microbiome composition, microbial metabolites, or neurochemical biomarkers should not be assumed to represent clinical benefit unless they are demonstrably associated with validated patient‐relevant outcomes. Therefore, gut–organ mechanisms should currently be interpreted as biologically plausible and potentially contributory pathways rather than established causal explanations for clinical efficacy [79].

## 3. Applications of Probiotics in Therapy

The mechanistic evidence summarized above provides a rationale for investigating probiotics as therapeutic or preventive interventions; however, biological plausibility does not necessarily translate into clinical benefit. Human studies are therefore required to determine whether experimentally observed effects on pathogen inhibition, intestinal barrier function, immune regulation, or microbial composition produce meaningful patient‐relevant outcomes. The clinical evidence discussed in this section is consequently considered separately from mechanistic evidence, with particular attention to the consistency of findings, study design, strain and dose specificity, and limitations in the available human data.

### 3.1. Treatment and Prevention of Antibiotic‐Associated Diarrhea (AAD)

AAD is a common adverse effect linked to the use of antibiotics, particularly prevalent among hospitalized patients who are treated with broad spectrum antibiotics [108]. Probiotics have increasingly emerged as a viable option for offering a safe method to mitigate the negative effects of antibiotics on GIT health. Mechanisms of action of probiotics against AAD include: (i) enhancing immune function; (ii) strengthening gut barrier function; (iii) generating antimicrobial agents; (iv) modifying the gut microbiome; (v) enhancing water absorption through elevating the expression of aquaporin; and (vi) reducing opportunistic pathogens [109].

Evidence for probiotics in the prevention of AAD is relatively substantial, but the magnitude and consistency of benefit vary according to population, probiotic formulation, dose, and study characteristics. A Cochrane review of 33 randomized controlled trials involving 6352 children found that probiotics reduced the risk of AAD compared with control (RR 0.45; 95% CI 0.36–0.56; *I* ^2^ = 57*%*), with moderate‐certainty evidence; however, substantial heterogeneity remained across studies [110].

Evidence from adult populations is similarly supportive but heterogeneous. A systematic review and meta‐analysis of 42 studies involving 11,305 adults reported a 37% reduction in AAD risk (RR 0.63; 95% CI 0.54–0.73), with moderate overall certainty, whereas subgroup analyses indicated that effects varied according to probiotic species and dose [111]. These findings suggest that the apparent benefit of probiotics should not be generalized to all strains or formulations. Importantly, evidence in older adults is less consistent; an earlier meta‐analysis found no significant overall preventive effect in elderly patients, whereas subsequent evidence suggested that probiotic administration within the first 48 hours of antibiotic treatment may be associated with greater preventive benefit in older adults [108, 112]. Thus, although probiotics can reduce AAD risk in selected populations, the optimal strain, dose, timing, and duration remain incompletely defined. Clinical heterogeneity, differences in baseline AAD risk, antibiotic exposure, probiotic composition, and outcome definitions further limit direct comparison between trials. Consequently, the evidence supports probiotics as a potentially useful adjunct for AAD prevention rather than a uniformly effective intervention across all antibiotic‐treated patients.

### 3.2. Management of IBD and IBS

IBS is defined by the presence of abdominal discomfort and changes in bowel habits. Symptoms often include abdominal bloating and distension. In contrast, IBD, principally ulcerative colitis and CD, is marked by persistent immune mediated inflammation of GIT [113]. The clinical features of IBD include hemorrhagic diarrhea, abdominal discomfort, weight reduction, and fatigue. Additionally, certain patients may present with extraintestinal symptoms, which can include skin lesions, respiratory issues, or arthritis [114].

Probiotics exert their effects in alleviating IBS through several mechanisms. One key mechanism involves the upregulation of the Mucin2 gene, which promotes the production and secretion of mucin from human intestinal cells. Additionally, probiotics that contain MUB enhance their attachment to intestinal cells. They also compete with, exclude, and displace pathogenic bacteria at the same adhesion sites, thereby preventing the invasion of these harmful microorganisms [55].

Probiotic mechanisms in the management IBD include: the suppression of harmful bacteria on IECs, enhancement of the intestinal cell barrier through promotion of mucus and AMPs production, modification of the mucosal immune response by influencing natural killer cell activity and the nuclear factor kappa‐B signaling pathway, and the induction of T‐cell apoptosis, which leads to an increase in the secretion of anti‐inflammatory cytokines (such as IL‐10 and TGF‐*β*) while simultaneously decreasing the levels of proinflammatory cytokines such as TNF‐*α*, IFN‐*γ*, and IL‐8 [55].

In vitro studies using IEC models have shown that selected *Bifidobacterium* strains can modulate inflammatory responses, including the expression of inflammatory genes and cytokine production. In patients with IBD, clinical evidence suggests that some probiotic interventions may modify the intestinal microbiota and provide clinical benefits, particularly in ulcerative colitis, although these effects appear to be strain‐specific and remain heterogeneous [113].

Evidence for probiotics in IBS and IBD is more heterogeneous than that for AAD, with effects varying according to probiotic formulation and study characteristics. In IBS, a systematic review and meta‐analysis of 20 studies involving 3011 patients found that probiotics improved global IBS symptom response (RR = 1.401; 95% CI: 1.182–1.662; *p* < 0.001) and quality of life (SMD = 0.286; 95% CI: 0.154–0.418; *p* < 0.001), whereas high‐dose (≥ 10^10^ CFU/day) and multistrain preparations also improved abdominal pain; however, substantial heterogeneity limits the generalizability of these findings [115]. In IBD, a 2024 overview and updated meta‐analysis of 45 randomized controlled trials found increased clinical remission in ulcerative colitis (OR = 2.00; 95% CI: 1.28–3.11), but not in CD (OR = 1.61; 95% CI: 0.21–12.50), with low overall certainty and no significant improvement in endoscopic outcomes [116]. These findings indicate that probiotic effects are disease and formulation‐dependent and should not be generalized across probiotic preparations. Moreover, changes in microbiota composition or findings from in vitro HT‐29 models do not necessarily predict clinical benefit, as these outcomes do not fully capture host‐specific factors, intestinal microbial interactions, disease activity, or patient‐relevant endpoints. Therefore, probiotic efficacy should be evaluated using clinically meaningful outcomes rather than microbiological or mechanistic changes alone.

### 3.3. Role in Preventing Allergic Reactions and Enhancing Immune Function

An allergy is characterized by a hypersensitive reaction that occurs due to an immunological response to a particular antigen, referred to as an allergen. Allergen interacts with innate immune cells. Continuous exposure to an allergen leads to the activation of mast cells and basophils, which subsequently release allergic mediators. This process can produce a variety of symptoms, including sneezing, itchy rashes, and in severe cases, shortness of breath and anaphylaxis [117].

Probiotics inhibit the invasion of harmful microorganisms by competing for binding sites on host′s epithelial cells. Furthermore, probiotic bacteria play a role in the maturation of DCs, promoting the production of anti‐inflammatory cytokines such as IL‐10. They are also important in managing allergic diseases through enhancement of mucosal immunoglobulin A (IgA) levels, as well as the stimulation of allergen‐specific T and B cells [118]. The role of probiotics in mitigating allergies involves several mechanisms: the regulation of the equilibrium between T helper cells (Th1) and Th2, the activation of Treg cells, and the modulation of the production of IgE, IgA, and IgM [55].

Evidence for probiotics in pediatric atopic dermatitis remains heterogeneous and appears to depend on probiotic formulation, timing of intervention, and characteristics of the target population. For treatment of established atopic dermatitis, a systematic review and meta‐analysis of nine randomized controlled trials involving 1000 children found that probiotic supplementation significantly reduced SCORAD scores in the continuous‐outcome analysis (MD = −4.24; 95% CI: −7.78 to −0.71; *p* = 0.002; *I* ^2^ = 71*%*). However, the analysis of dichotomous outcomes did not demonstrate a significant effect, and the authors emphasized the limited quantity and quality of the available evidence [119]. In contrast, a meta‐analysis of 37 randomized controlled trials involving 6131 participants found that probiotics reduced the risk of developing atopic dermatitis (RR = 0.83; 95% CI: 0.73–0.94; *I* ^2^ = 65.2*%*), with subgroup analyses suggesting that preventive effects varied according to timing of administration and probiotic formulation, including maternal and infant supplementation and preparations containing *Lactobacillus rhamnosus* or multiple strains [120].

Together, these findings suggest potential benefits of probiotics for both prevention and reduction of disease severity; however, they do not establish a uniform class effect. Differences in probiotic strains and formulations, timing of administration, patient characteristics, outcome measures, and substantial between‐study heterogeneity limit generalizability. Further well‐designed trials are needed to determine the optimal strains, doses, timing, and duration of intervention.

The selection of probiotic microorganisms for clinical application should consider key characteristics, including safety, viability, tolerance to gastrointestinal conditions, antimicrobial activity, adhesion to intestinal surfaces, and stability during storage (Figure 4).

**Figure 4:**
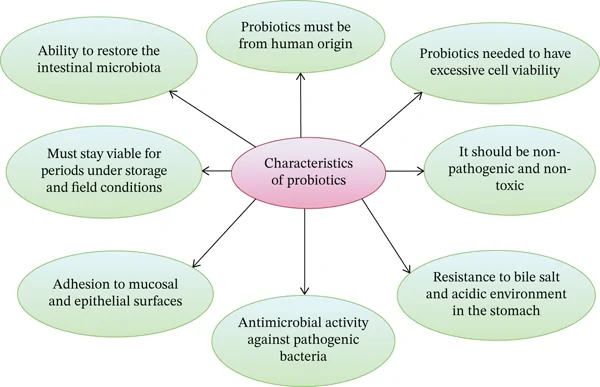
Key characteristics and selection criteria for probiotic microorganisms. Created by the authors using Microsoft Paint and Microsoft Word (Microsoft Corporation, Redmond, Washington, United States).

The central node (“Characteristics of Probiotics”) is surrounded by eight criteria, each representing a requirement for a candidate strain to be considered suitable for probiotic use. These properties can be grouped into three broad categories: (i) safety and origin, (ii) functional and physiological performance in the host, and (iii) technological and manufacturing robustness.

Biological processes and criteria illustrated:

1. Human origin: Probiotic strains intended for human use are typically expected to be isolated from human sources (e.g., human intestinal microbiota or breast milk), which is considered advantageous for colonization, adaptation to the human gastrointestinal environment, and safety.
2. Excessive cell viability: The preparation must contain a sufficient number of viable cells to confer a health benefit, consistent with the definition of probiotics as “live microorganisms which, when administered in adequate amounts, confer a health benefit on the host.”
3. Nonpathogenic and nontoxic: The strain must be safe for consumption, free from virulence determinants, and must not produce harmful metabolites or toxins. Safety assessment typically includes evaluation of hemolytic activity, antibiotic‐resistance gene transfer potential, and toxigenicity.
4. Resistance to bile salts and acidic environments in the stomach: To reach the colon in a viable state, probiotic strains must survive passage through the acidic gastric environment (low pH) and tolerate bile salts in the small intestine, both of which are major barriers to survival and colonization.
5. Antimicrobial activity against pathogenic bacteria: Probiotic strains may inhibit pathogenic microorganisms through the production of antimicrobial substances (e.g., organic acids, bacteriocins, hydrogen peroxide, and diacetyl) and through competitive exclusion mechanisms.
6. Adhesion to mucosal and epithelial surfaces: Adhesion to IECs and mucus is considered an important property because it facilitates transient colonization, prolongs residence time in the gut, and contributes to competitive exclusion of pathogens.
7. Viability during storage and under field conditions: Probiotic products must maintain adequate viability and functional activity throughout processing, storage, distribution, and use. This property is closely related to formulation and stabilization technologies, such as lyophilization, encapsulation, and appropriate packaging.
8. Ability to restore the intestinal microbiota: Probiotic strains should contribute to the restoration or maintenance of a balanced gut microbial community, particularly following disruption such as that caused by antibiotic therapy, infection, or other stressors.

The evidence supporting probiotic interventions varies substantially according to the experimental model, probiotic strain, and clinical condition investigated. Although in vitro and animal studies provide important insights into antimicrobial activity, intestinal barrier protection, and immunomodulatory mechanisms, these findings do not necessarily establish clinical efficacy in humans. Human clinical trials provide more direct evidence of therapeutic benefits, although outcomes remain dependent on strain characteristics, dosage, population, and disease context. An overview of the available experimental and clinical evidence, including observed outcomes, proposed mechanisms, evidence levels, and translational limitations, is presented in Table 3.

**Table 3:** Evidence hierarchy, key outcomes, and proposed mechanisms of probiotic interventions across experimental and clinical studies.

Probiotic species/strain	Experimental model	Key outcome(s)	Proposed mechanism(s) of action	Evidence level	Translational gap/limitation	Reference
*Lactococcus lactis* (engineered strain)	In vitro (coculture)	Reduced *E. faecium* counts	Production and delivery of antimicrobial peptides (AMPs)	Level III (mechanistic/in vitro)	No in vivo or clinical confirmation	[121]
*Lactobacillus rhamnosus* GG	In vitro food‐contact surface model	Reduced adhesion of *E. coli* O157:H7 and other foodborne pathogens	Antiadhesive and biofilm‐displacement effects	Level III (mechanistic)	Evidence from stainless‐steel surface models does not establish inhibition of intestinal colonization or clinical benefit	[122]
*L. rhamnosus* GG and *Lactobacillus paracasei* IMPC2.1	In vitro epithelial cell models	Reduced inflammation and LPS‐induced epithelial barrier dysfunction	Modulation of inflammatory signaling, tight‐junction integrity, and autophagy	Level III (mechanistic)	Requires validation in animal and clinical studies	[123, 124]
*Lactococcus lactis* 368, *Lactobacillus curvatus* MBSa3 and *Lactobacillus sakei* MBSa1	In vitro pathogen biofilm assays	Strong inhibition of *Salmonella typhimurium*, *Listeria monocytogenes*, and *E. coli* O157:H7 biofilm formation	Competitive exclusion/coaggregation and production of inhibitory substances; effect not attributable solely to bacteriocin production	Level III (mechanistic/in vitro)	Activity demonstrated in food‐associated biofilm models; requires validation in complex gut ecosystems and in vivo/clinical settings	[125]
Probiotic interventions, including *L. rhamnosus* GG and other *Lactobacillus*, *Bifidobacterium*, and *Saccharomyces* strains	Human RCTs and systematic reviews/meta‐analyses in adults and children	Reduced risk of antibiotic‐associated diarrhea (AAD) in selected adult and pediatric populations	Potential microbiota modulation, competitive exclusion/adhesion interference, gut‐barrier support, and immunomodulatory effects	Level I (clinical)	Effects vary according to strain, dose, antibiotic exposure, age, baseline risk, and population; the optimal strain, dose, timing, and duration remain incompletely defined	[111, 126]
*Lactobacillus reuteri* LMG P‐27481	Animal model (C57BL/6 mouse)	Reduced *C. difficile* colonization and toxin levels; attenuated intestinal inflammatory damage	Antimicrobial activity and modulation of inflammatory responses; potential effects on intestinal barrier function	Level II (preclinical)	Human efficacy and safety remain to be established	[127]
*Lactobacillus plantarum* BSGP201683 (G83)	Animal model (giant‐panda microbiota‐associated mice challenged with ETEC K88)	Reduced *E. coli* K88‐associated effects and improved intestinal barrier integrity, including increased expression of Claudin‐1, ZO‐1, and Occludin‐1	Modulation of intestinal microbiota and enhancement of epithelial barrier function	Level II (preclinical)	Human clinical studies are needed to determine strain‐specific efficacy and clinical relevance	[128]
*Lactobacillus plantarum* MB452	In vitro (Caco‐2 monolayer)	Increased TEER (trans‐epithelial electrical resistance) and enhanced intestinal epithelial barrier function	Increased expression/abundance of tight‐junction‐associated proteins, including ZO‐1, ZO‐2, and occludin	Level III (mechanistic)	TEER and tight‐junction changes in Caco‐2 cells do not establish clinically meaningful barrier improvement in humans	[129]
*Pediococcus pentosaceus* LI05	Animal model (C57BL/6 mouse model of *C. difficile* infection)	Improved survival and attenuated *C. difficile*‐associated colonic injury	Suppression of inflammatory responses and preservation of intestinal barrier integrity through modulation of ZO‐1, occludin, and claudin‐1	Level II (preclinical)	Human efficacy and clinical relevance remain unestablished	[130]
*Lactococcus lactis* IL1403 (recombinant)	In vitro (antimicrobial assays)	Recombinant IL1403 inhibited growth of pathogenic *E. coli* and *Salmonella*	Secretion of heterologous antimicrobial peptides (A3APO and Alyteserin‐1a)	Level III (mechanistic)	No in vivo confirmation; efficacy and safety of the engineered system require validation in appropriate biological models	[131]
*Lactobacillus johnsonii* MH‐68	Animal model (mouse)	Reduced *H. pylori* load in gastric mucosa and attenuated infection‐associated inflammatory changes	Antagonistic activity against *H. pylori*, interference with pathogen adhesion, and modulation of inflammatory responses	Level II (preclinical)	Clinical efficacy was not established by this animal study; strain‐specific clinical evidence requires consideration	[132]
*Bifidobacterium lactis* (LACT GB), *L. rhamnosus* (RHAM GB), and *Lactobacillus reuteri* (REUT GB)	Animal model (BALB/c mice with LPS‐induced inflammation)	Reduced inflammatory and oxidative responses and preserved intestinal and brain tissue integrity; *B. lactis* supplementation was also associated with reduced anxiety‐like behavior	Immunomodulation involving inflammatory and oxidative‐stress responses along the gut–brain axis	Level II (preclinical)	Causal mechanisms and clinical relevance remain to be established	[133]
Multistrain Protexin containing *Lactobacillus*, *Bifidobacterium*, and *Streptococcus thermophilus* strains	Human randomized, double‐blind, placebo‐controlled trial in children with chronic constipation	Increased stool frequency and improved stool consistency after 4 weeks; short‐term improvements in abdominal pain and fecal incontinence were also observed	Effects on gastrointestinal function; microbiota‐mediated mechanisms were not directly assessed	Level I (clinical)	Strain‐specific contributions and microbiota‐mediated mechanisms remain unclear; long‐term clinical outcomes require further evaluation	[134]
*Akkermansia muciniphila*	Animal and human studies	Improved metabolic and intestinal phenotypes in animal models; clinical effects in overweight/obese individuals with type 2 diabetes were dependent on baseline intestinal *A. muciniphila* abundance	Amuc_1100‐mediated host signaling, with immunomodulatory and intestinal barrier‐related effects	Level II–I (preclinical to clinical)	Clinical effects appear context‐dependent and require confirmation in larger, longer term studies	[135]
*Lactobacillus plantarum* EM	In vitro cholesterol‐assimilation assays and animal model (hypercholesterolemic rats)	Cholesterol removal in vitro; fermented juice containing *L. plantarum* EM reduced serum total cholesterol, triglycerides and LDL‐cholesterol in rats	Cell‐surface cholesterol binding/assimilation and possible bile‐salt hydrolase activity; in rats, modulation of cholesterol synthesis, uptake and excretion	Level II–III (preclinical/mechanistic)	No human clinical evidence establishing serum lipid‐lowering efficacy	[136]
Multispecies probiotic containing *Lactobacillus acidophilus*, *Bifidobacterium bifidum*, *B. lactis*, and *B. longum*; inulin‐based synbiotic	Human randomized, double‐blind, placebo‐controlled trial in adults with prediabetes	Reduced prevalence of metabolic syndrome; probiotic supplementation also reduced hyperglycemia, hypertension and low HDL‐cholesterol prevalence	Proposed effects through gut microbiota modulation and metabolic signaling; specific SCFA–GLP‐1 mechanisms were not directly assessed	Level I (clinical)	Strain‐specific contributions and microbiota‐mediated mechanisms remain unclear; long‐term clinical effects require further evaluation	[137]

*Note:* Evidence level: Level I (Clinical): Evidence from human randomized controlled trials (RCTs), meta‐analyses of RCTs, or well‐designed human intervention studies. Level II (Preclinical): Evidence from animal models or other in vivo experimental studies. Level III (Mechanistic): Evidence derived primarily from in vitro, cell‐based, biochemical, or other experimental models. When evidence spans multiple levels without direct human mechanistic confirmation, combined designations (e.g., Level II–III) may be used. Study design/model: Specifies the experimental system (in vitro, animal model, human RCT, and meta‐analysis) from which the evidence was derived. Translational gap: Refers to limitations in extrapolating mechanistic or preclinical findings to the complex human gastrointestinal environment and clinical settings, or to incomplete definition of optimal clinical parameters for human studies.

## 4. Prebiotics in Reshifting Dysbiosis: Mechanisms of Action of Prebiotics

The major mechanisms can be grouped into modulation of gut microbiota, production and signaling of SCFAs, regulation of intestinal barrier and mucus function, and immunomodulation. These mechanisms are interconnected rather than independent; changes in microbial composition may alter metabolite production, which can subsequently influence epithelial, immune, and metabolic pathways.

### 4.1. Modulation of Gut Microbiota

Prebiotics are selectively utilized by intestinal microorganisms and can modify the composition and metabolic activity of the gut microbiota. Established prebiotic substrates, including inulin‐type fructans (ITFs), FOS, and GOS, can promote the growth or activity of microorganisms capable of utilizing these substrates, including members of the genera *Bifidobacterium* and *Lactobacillus* [138]. Through this selective microbial utilization, prebiotics may alter microbial community structure and enhance the production of microbial metabolites, particularly SCFAs, with potential effects on host physiology [139].

Although prebiotics can modify gut microbial composition, their effects are not uniformly selective or predictable across individuals. The magnitude and direction of microbial responses depend on the chemical structure and degree of polymerization of the prebiotic, the characteristics of the intervention, and the composition and functional capacity of the resident microbiota. In particular, increases in *Bifidobacterium* and *Lactobacillus* reported with ITFs, FOS, or GOS should not be interpreted as universal responses, because other taxa may also utilize these substrates and individual microbiomes differ in their carbohydrate‐utilization capacities. Moreover, changes in the relative abundance of specific bacterial taxa do not necessarily indicate improved overall microbiota function or restoration of a dysbiotic community. Thus, current evidence supports prebiotics as context‐dependent modulators of microbial community structure and activity, with the extent and consistency of microbiota modulation depending on both substrate characteristics and host‐specific microbial ecology [140, 141].

### 4.2. SCFA Production and Signaling

Gut microbiota ferment prebiotics into SCFAs, principally acetate, propionate, and butyrate, primarily in the colon. These metabolites have both metabolic and signaling functions. Butyrate serves as an important energy substrate for colonocytes, whereas acetate and propionate undergo distinct metabolic fates in peripheral tissues and the liver. Propionate can contribute to hepatic gluconeogenic pathways, whereas acetate contributes to systemic energy metabolism. SCFAs can interact with host receptors, including free fatty acid receptor 2 (FFAR2) and free fatty acid receptor 3 (FFAR3), and influence enteroendocrine signaling. These interactions may stimulate the secretion of glucagon‐like peptide‐1 (GLP‐1) and peptide YY (PYY), thereby contributing to the regulation of glucose homeostasis, appetite, gastrointestinal motility, and metabolic physiology [4, 142].

Although SCFA production is a well‐established consequence of microbial fermentation of many prebiotic substrates, the magnitude and composition of the SCFA response are highly context‐dependent. The relative production of acetate, propionate, and butyrate varies according to the type and characteristics of the prebiotic, microbial community composition, and intestinal transit time [141]. Moreover, increased fecal SCFA concentrations should not necessarily be interpreted as increased total SCFA production or systemic exposure, because SCFAs are extensively absorbed and metabolized along the GIT, including substantial utilization by colonocytes and subsequent metabolism in peripheral tissues [141].

Evidence for SCFA‐mediated signaling through free fatty acid receptor 2 (FFAR2/GPR43) and free fatty acid receptor 3 (FFAR3/GPR41) provides a plausible mechanism linking microbial fermentation with host metabolic and enteroendocrine responses. However, mechanistic evidence for receptor activation should not be equated with clinically meaningful effects of dietary prebiotic supplementation on GLP‐1, PYY, glucose regulation, or appetite in humans. Similarly, systemic effects attributed to circulating SCFAs require careful interpretation because a substantial proportion of SCFAs is utilized within the GIT, particularly by colonocytes, whereas the fraction entering the portal and systemic circulation is further subject to hepatic and peripheral tissue metabolism. Thus, although SCFAs are well‐established metabolic products of microbial fermentation, the extent to which prebiotic‐induced changes in SCFA production translate into systemic physiological or clinical benefits remains dependent on microbial metabolism and host context [142].

### 4.3. Intestinal Barrier and Mucus Regulation

Prebiotics may support intestinal barrier integrity indirectly by altering gut microbial composition and metabolic activity and by promoting the production of microbial metabolites. These changes may influence mucus production and the composition and function of the intestinal mucus layer, which forms an important protective interface between the gut microbiota and intestinal epithelium [143]. Fermentation‐derived SCFAs, particularly butyrate, may contribute to epithelial barrier maintenance by influencing the expression and organization of tight‐junction proteins, including ZO‐1, occludin, and claudins. These effects may help maintain epithelial integrity and intestinal homeostasis. Through microbiota‐ and metabolite‐mediated effects, prebiotics may therefore support the mucus epithelial barrier and contribute to the regulation of intestinal permeability [4, 144].

Evidence supporting a barrier‐protective effect of prebiotics is biologically plausible but remains heterogeneous across experimental models and substrates. Mechanistic studies have suggested effects on mucus production, tight‐junction regulation, and epithelial permeability; however, much of this evidence derives from in vitro and animal models. Human evidence, particularly regarding intestinal permeability, remains limited and heterogeneous, making the translational relevance of these mechanisms uncertain [145].

Moreover, changes in ZO‐1, occludin, or claudin expression should not be considered direct evidence of improved intestinal barrier function, as changes in tight‐junction protein expression do not necessarily correspond to functional changes in epithelial permeability. The effects of prebiotics may also be mediated indirectly through changes in microbial composition and metabolite production, making it difficult to attribute barrier responses to the prebiotic substrate itself or to a specific metabolite such as butyrate. In addition, responses may vary according to prebiotic type, dose, baseline microbiota, and host physiological state. Thus, current evidence suggests that prebiotics may contribute to mucus–epithelial barrier regulation; however, evidence for this effect remains less consistent and less clinically established than the well‐characterized role of prebiotic fermentation in shaping the gut microbiota and promoting SCFA production [146].

### 4.4. Immunomodulation

Prebiotics may influence immune function indirectly by altering gut microbial composition and the production of fermentation‐derived metabolites. Among these metabolites, butyrate has been shown in experimental systems to modulate inflammatory signaling, including the production of proinflammatory cytokines such as interleukin (IL)‐6 and IL‐12. Butyrate may also influence DC function and promote Treg differentiation in experimental systems, potentially contributing to immune tolerance and regulation of intestinal inflammation. Butyrate may additionally influence macrophage function and polarization, with effects that can contribute to the regulation and resolution of inflammation. Other SCFAs, including propionate and acetate, have also been reported to modulate cytokine production and inflammatory signaling pathways [4].

Although these findings provide plausible mechanisms through which prebiotics may influence intestinal immune responses, the strength of evidence differs substantially among the proposed effects. Much of the mechanistic evidence for SCFA‐ and particularly butyrate‐mediated effects on cytokine production, DC function, Treg differentiation, and macrophage polarization derives from in vitro and animal studies, often involving direct exposure to SCFAs rather than prebiotic supplementation in humans [147].

Consequently, these findings cannot be assumed to represent the effects of consuming a prebiotic in humans, where SCFA production and downstream immune responses may vary according to substrate characteristics, intervention dose, baseline microbiota composition, and host characteristics. Furthermore, immunological responses to SCFAs are context‐dependent, and changes in inflammatory biomarkers do not necessarily translate into clinically meaningful improvements in intestinal inflammation. Thus, prebiotics have a biologically plausible indirect immunomodulatory mechanism mediated partly through microbial metabolites; however, evidence linking prebiotic‐induced changes in microbiota composition or SCFA production to consistent immune benefits in humans remains limited and heterogeneous [147].

## 5. Applications of Prebiotics in Therapy

The following sections examine the principal clinical applications of prebiotics, with emphasis on the strength and limitations of human evidence. Although the mechanisms described above provide biological plausibility, evidence from in vitro and animal studies cannot be assumed to translate directly into clinical benefit. Accordingly, clinical effects are evaluated based on human studies, with particular attention to consistency, study design, population characteristics, and sources of heterogeneity. Effects that vary according to baseline microbiota, host characteristics, dose, or disease stage are considered context dependent, whereas findings reproduced across diverse populations are regarded as more broadly conserved.

### 5.1. Regulation of Bowel Movements and Prevention of Constipation

Constipation is characterized by disturbances in gastrointestinal (GIT) motility, alterations in gut microbiota composition, and changes in CNS processing. Its etiology is multifactorial, with gut microbiota dysbiosis recognized as a significant contributor alongside inadequate fluid intake, insufficient dietary fiber, excessive alcohol consumption, and endocrine, neurological, or physiological conditions [148]. Prebiotics stimulate the proliferation of *Bifidobacterium* spp., modulate immune responses, and suppress the growth of harmful gut microbiota. SCFAs produced during prebiotic fermentation supply energy to the colonic epithelium, alleviate intestinal inflammation, and influence gut motility, providing the mechanistic basis for prebiotic use in constipation [149].

Human evidence for prebiotics in constipation is comparatively stronger than for many other indications reviewed here, but it remains heterogeneous. Clinical trials differ substantially in the type of prebiotic used, dose, intervention duration, participant characteristics, and outcome definitions. Moreover, benefits are not consistently observed across all endpoints; some studies report improvements in stool frequency or consistency without corresponding effects on other measures of bowel function [150, 151].

### 5.2. Reducing Inflammation in Conditions Such as IBD

Prebiotics promote the proliferation of beneficial microorganisms that can compete with pathogenic species and increase the production of SCFAs. SCFAs can exert immunomodulatory effects and contribute to the regulation of intestinal inflammatory responses and cytokine production [152].

Mechanistic support for SCFA‐mediated immunomodulation is strong from in vitro and animal models, but clinical translation in IBD is selective rather than general. A systematic review and meta‐analysis found that, in ulcerative colitis, FOS kestose was effective for induction of remission (RR 2.75, 95% CI 1.05–7.20), whereas germinated barley foodstuff showed a trend toward preventing relapse. In contrast, the evidence for other prebiotics in ulcerative colitis is inconsistent and of very low certainty. Oligofructose‐enriched inulin and lactulose did not demonstrate significant benefit for induction of remission, whereas oligofructose‐enriched inulin, oat bran, and *Plantago ovata* did not demonstrate significant benefit for maintenance of remission. In CD, oligofructose‐enriched inulin and lactulose were no different from controls for induction of remission, whereas FOS was no different from control for maintenance of remission. Overall, these findings suggest that the effects of prebiotics in IBD are prebiotic‐ and disease‐specific rather than indicative of a general class effect, with the overall certainty of evidence remaining very low. Prebiotics should therefore be considered potential adjunctive interventions requiring further evaluation, rather than established therapies for IBD [153, 154].

### 5.3. Supporting Metabolic Health by Reducing the Risk of Obesity and Type 2 Diabetes

Metabolic syndrome is characterized by metabolic abnormalities including insulin resistance, dyslipidemia, hypertension, and abdominal obesity, which collectively increase the risk of cardiovascular disease and type 2 diabetes mellitus [155]. The modulation of enteroendocrine function by prebiotics and their fermentation products, particularly SCFAs, is proposed to contribute to their effects on glucose and lipid homeostasis. Prebiotic‐induced changes in mucus composition and mucin turnover may also help maintain intestinal barrier integrity and attenuate obesity‐associated inflammation. Some prebiotics may additionally interact directly with host tissues to promote barrier function and improve glucose metabolism, although the relevance of these effects in humans remains difficult to establish. Prebiotics may further enhance endogenous production of intestinal glucagon‐like peptide‐2 (GLP‐2), which reinforces gut barrier function through upregulation of critical tight‐junction proteins in the intestinal epithelium [156].

A GRADE‐assessed meta‐analysis of 33 trials involving 1346 participants found that ITFs significantly reduced fasting blood glucose (WMD −0.60 mmol/L; high‐certainty evidence), HbA1c (WMD −0.58%; high‐certainty evidence), fasting insulin (WMD −1.75 *μ*U/mL; low‐certainty evidence), and HOMA‐IR (WMD −0.69; low‐certainty evidence) in populations with prediabetes and type 2 diabetes. The authors identified a dose of 10 g/day administered for ≥ 6 weeks as being associated with improved glycemic outcomes in this population [157].

A separate meta‐analysis of 33 randomized controlled trials demonstrated that ITF intake significantly reduced blood glucose, total cholesterol, and triglycerides among participants with prediabetes or diabetes, whereas these effects were not observed in healthy, overweight/obese, or hyperlipidemic populations. Body weight was unaffected, and no overall significant effect on blood insulin was observed [158]. These findings indicate that the metabolic effects of ITF are context‐ and population‐dependent rather than representing a uniform class effect. Accordingly, ITF may be considered a potential adjunct to, rather than a replacement for, established metabolic interventions.

Different prebiotic substances vary in their chemical structures, fermentability, mechanisms of action, and health effects. Evidence from experimental and clinical studies indicates that their benefits may depend on the type and dose of prebiotic, the baseline gut microbiota composition, and the characteristics of the study population. A summary of commonly investigated prebiotic substances, their proposed mechanisms, reported health effects, and supporting evidence is presented in Table 4.

**Table 4:** Prebiotic substances, representative sources, microbiota effects, mechanisms, and evidence strength.

Prebiotic substance	Examples	Reported microbiota/metabolite effects	Proposed mechanisms	Evidence level	References
Inulin‐type fructans (ITF)	Inulin, fructooligosaccharides (FOS), oligofructose	Consistently increase *Bifidobacterium* abundance. Effects on other bacterial groups are less consistent. SCFA changes, including acetate, propionate and butyrate, have been reported but vary between studies.	ITF reaches the colon largely undigested, where it is preferentially fermented by carbohydrate‐utilizing bacteria. This promotes *Bifidobacterium* and other saccharolytic bacteria and increases microbial metabolites such as SCFAs.	Strong human evidence for increasing *Bifidobacterium*; moderate and variable evidence for SCFA changes. Effects depend on dose, chain length, baseline microbiota and host characteristics.	[159, 160]
Lactulose	Synthetic disaccharide of galactose and fructose	Promotes lactulose‐utilizing bacteria, including *Bifidobacterium* and *Lactobacillus* spp. in some human studies. Fermentation increases organic acids, SCFAs and hydrogen. The magnitude of these effects varies with dose and patient characteristics.	Lactulose is poorly absorbed in the small intestine and reaches the colon, where bacteria ferment it. Fermentation produces organic acids that lower colonic pH and support acid‐tolerant saccharolytic bacteria.	Moderate human evidence, with stronger evidence for effects on bowel function than for consistent changes in microbiota or metabolites.	[161]
*β*‐Glucans	Oat‐ and barley‐derived *β*‐glucans	Alter gut microbial composition and fermentation activity. Human studies have reported changes in bacterial taxa and increases in acetate and other SCFAs, but findings are not consistent across studies.	Gut bacteria degrade and ferment *β*‐glucans. Their effects depend on molecular structure and accessibility to microbial enzymes. Fermentation produces SCFAs that can influence intestinal and metabolic signaling.	Moderate and heterogeneous human evidence. Effects vary with *β*‐glucan source, molecular weight, structure, dose, food matrix and baseline microbiota.	[162, 163]
Resistant starch (RS)	RS1–RS5	Increases some starch‐fermenting and SCFA‐producing bacteria, including *Ruminococcus*, *Agathobacter*, *Faecalibacterium*, and *Bifidobacterium* in some human interventions. Increased SCFA production, particularly butyrate, has also been reported.	Resistant starch escapes digestion in the small intestine and reaches the colon. There, bacteria ferment it directly and exchange fermentation products through microbial cross‐feeding, leading to SCFA production.	Moderate and heterogeneous human evidence. Effects depend on RS type, structure, dose, intervention duration, baseline microbiota and host characteristics.	[164, 165]
Pectin and pectic oligosaccharides (POS)	Citrus pectin, apple pectin, pectic oligosaccharides	Stimulate pectin‐degrading and other carbohydrate‐utilizing bacteria. Fermentation studies report increased acetate and enrichment of bacteria such as members of *Ruminococcaceae*, *Bacteroides*, *Lachnospira*, and *Faecalibacterium*.	Pectin is degraded and fermented by intestinal microorganisms. The resulting metabolites, including acetate, propionate and butyrate, may affect host intestinal signaling. Microbial cross‐feeding can further increase fermentation products.	Emerging evidence, mainly from in vitro studies. Effects depend on pectin structure, degree of methyl esterification and molecular weight. Evidence from human studies remains limited.	[166]

## 6. Challenges of Using Probiotics and Prebiotics

### 6.1. Variability in Clinical Outcomes and Host Responses

Interindividual variability in gut microbiota composition, genetics, diet, and lifestyle presents an additional challenge for standard therapeutic approaches. Although personalized microbiota‐targeted interventions show promise, the lack of standardized microbiome profiling methods and clinical implementation frameworks limits their widespread application [68, 167].

#### 6.1.1. Influence of Baseline Microbiota Composition

The pre‐existing gut microbial community can strongly influence probiotic engraftment. Maldonado‐Gómez et al. [168] demonstrated that *B. longum* AH1206 stably persisted in approximately 30% of healthy individuals for at least 6 months, with successful engraftment associated with lower baseline abundance of resident *B. longum* and underrepresentation of specific carbohydrate‐utilization genes in the pretreatment microbiome.

Suez et al. [169] reported that, following antibiotic treatment, administration of a multispecies probiotic preparation was associated with delayed and incomplete recovery of the indigenous gut microbiota, whereas autologous fecal microbiota transplantation promoted more rapid and near‐complete microbiota reconstitution. These findings highlight that probiotic administration does not necessarily accelerate recovery of the native microbiome after antibiotic‐induced disruption and that effects may depend on the intervention and the individual′s microbiome context. Baseline gut microbiota composition can substantially influence individual responses to prebiotic interventions. Individuals with lower baseline abundance of *Bifidobacterium* may exhibit greater increases in bifidobacterial abundance following prebiotic supplementation, whereas baseline microbial characteristics and habitual dietary fiber intake may also contribute to variability in SCFA responses [170–172].

The influence of baseline microbiota composition on probiotic persistence and activity illustrates an important distinction between mechanisms that may be broadly conserved and those whose effects depend strongly on host and microbial context. Although processes such as competitive exclusion, antimicrobial metabolite production, and modulation of epithelial and immune functions represent recurring mechanisms of probiotic action, their magnitude and functional consequences may vary according to probiotic strain, resident microbiota, host physiology, pathogen characteristics, and disease state. This distinction provides a framework for interpreting both the consistency and variability of probiotic responses across experimental and clinical settings. Accordingly, the major mechanisms discussed in this review are classified as either relatively broadly conserved or context dependent, as summarized in Table 5.

**Table 5:** Classification of gut microbiota–host interaction mechanisms by reproducibility and context dependence.

Mechanism type	Examples	Reproducibility	Implication
Broadly conserved	SCFA production, microbial bile‐acid transformation, and core epithelial barrier interactions	Observed across diverse experimental and/or human contexts, although magnitude may vary	Represents fundamental mechanisms underlying probiotic–host or microbiota interactions
Context‐dependent	Probiotic colonization, competitive exclusion, immunomodulation, and tryptophan‐metabolite signaling	Varies according to baseline microbiota, probiotic strain, host characteristics, and disease state	Effects require consideration of host and microbial context and may benefit from stratification

*Note:* “Broadly conserved” refers to mechanisms that are repeatedly observed across diverse probiotic strains or host contexts and should not be interpreted as implying identical magnitude or clinical efficacy in all individuals. “Context‐dependent” refers to mechanisms whose occurrence, magnitude, or functional consequences are substantially influenced by strain, host, microbiota, or disease characteristics.

The proposed framework (Table 5) distinguishes broadly conserved from context‐dependent microbiome mechanisms and yields two testable predictions that are supported by existing evidence. First, mechanisms that are broadly conserved, such as microbial fermentation of prebiotic substrates to SCFAs, should show greater reproducibility across individuals than mechanisms that depend strongly on the recipient microbiome. However, reproducibility should not be interpreted as uniformity of effect, because the magnitude and profile of SCFA responses may vary according to substrate characteristics, dose, baseline microbiota composition, habitual dietary intake, and host physiology. Human intervention studies and meta‐analyses generally support the capacity of prebiotic interventions to modify gut microbial composition and SCFA production, while also demonstrating variability in the magnitude and direction of individual responses [36, 173].

Second, context‐dependent mechanisms such as probiotic colonization are expected to depend more strongly on baseline ecological conditions. Studies of strain‐specific probiotic engraftment have shown that probiotic colonization can vary among individuals according to baseline host and microbiome characteristics, whereas differences in resident microbial communities have also been associated with probiotic persistence [174, 175]. Thus, probiotic persistence should not be regarded as a universal property of supplementation but rather as an outcome shaped by interactions between the administered strain and the recipient′s resident microbiome. This distinction provides a useful heuristic for evaluating microbiome interventions: Broadly conserved metabolic functions may be expected to show relatively reproducible effects, whereas ecological processes such as strain engraftment are more likely to exhibit substantial interindividual variability.

#### 6.1.2. Host Genetics and Immune Status

Genetic background may shape host–microbe interactions through variation in host genes involved in microbial sensing and immune regulation, including PRRs such as NOD2. NOD2 recognizes bacterial peptidoglycan‐derived molecules, and experimental evidence indicates that some probiotic *Lactobacillus* strains can generate NOD2‐stimulating ligands that contribute to intestinal immune and barrier regulation. These findings provide a plausible mechanistic basis for host genetic variation to contribute to interindividual differences in probiotic responses, although direct evidence linking specific NOD2 variants to differential clinical responses to probiotics remains limited [176, 177].

#### 6.1.3. Dietary Patterns and Environmental Factors

Diet is a major modulator of gut microbiota composition and activity, influencing substrate availability for microbial metabolism. Healey et al. [171] demonstrated that habitual dietary fiber intake influences the gut microbiota response to an ITF prebiotic, with individuals consuming a high‐fiber diet showing a greater microbiota response than those with low habitual fiber intake; increases in *Bifidobacterium* were observed in both groups. However, responses to prebiotic supplementation are not determined by habitual fiber intake alone. Baseline microbiota composition may also contribute to interindividual variability in response.

For example, Chung et al. [178] reported that the relative abundance of *Prevotella* at baseline was associated with differential responses to wheat‐bran arabinoxylan‐oligosaccharide supplementation, whereas Hoffmann et al. [179] similarly found that baseline intestinal microbiota composition influenced responses to a dietary fiber intervention. These findings suggest that prebiotic effects may depend on the ecological characteristics of the resident microbiota in addition to the amount and type of fiber consumed.

#### 6.1.4. Strain‐Specific Therapeutic Effects of Probiotics

Therapeutic effects are often strain‐specific, as individual strains exhibit distinct functional traits such as metabolite production, epithelial adhesion, and immunomodulatory activity. Clinical benefits demonstrated by one strain cannot be generalized to other strains within the same species [180].

#### 6.1.5. Clinical Heterogeneity

Significant heterogeneity exists in study design, dosing regimens, intervention duration, and outcome assessment making it difficult to compare results across studies. It further complicates data interpretation and restricts meta‐analytical integration and evidence synthesis, highlighting the need for standardized protocols and well‐controlled trials to generate reproducible and clinically meaningful evidence [181, 182].

### 6.2. Safety Consideration

Prebiotics are generally regarded as safe; however, gastrointestinal adverse effects, particularly diarrhea, bloating, and flatulence, may occur with higher doses, reflecting the fermentability and osmotic effects of these carbohydrates [183]. Probiotic‐associated bacteremia and fungemia are rare but documented complications, particularly among patients with severe underlying illness or compromised intestinal barriers. A systematic review identified 60 case reports and 7 case series comprising 93 patients; however, the evidence was derived predominantly from case reports and case series, limiting estimates of incidence and causal inference [184]. Subsequent evidence has shown that strain‐level microbiological or genomic comparison can strengthen attribution of bloodstream infections to an administered probiotic strain; in ICU patients, whole‐genome analyses provided evidence linking bloodstream *Lactobacillus* isolates to the administered probiotic strain [185].

### 6.3. Challenges in Translating Findings to Human Clinical Outcomes

Although mechanistic and preclinical studies are abundant, rigorous human clinical trials remain limited. Moreover, commonly used animal models do not fully replicate the complexity of the human gut microbiota, host–microbe interactions, immune responses, and environmental influences, thereby reducing the predictive validity of preclinical findings for clinical outcomes [186, 187].

### 6.4. Mechanistic Gaps

Several mechanisms have been proposed, including modulation of immune responses, enhancement of epithelial barrier integrity, and production of bioactive metabolites. However, the systemic effects of gut microbiota on distant organs remain incompletely understood, limiting a comprehensive understanding of host–microbiome interactions and contributing to difficulties in interpreting interindividual variability [188].

### 6.5. Regulatory and Commercial Challenges

Probiotics and synbiotics are often classified as dietary supplements rather than therapeutic agents, resulting in variability in regulatory oversight, labeling accuracy, and quality control. This classification restricts their formal evaluation within clinical guidelines and limits broader clinical adoption in evidence‐based medicine [189].

### 6.6. Technological and Formulation Constraints

Sustaining microbial viability, genetic stability, and functional activity during processing, storage, and GI transit remains a significant technical challenge. In addition, the optimization of synbiotic formulations‐including appropriate strain‐substrate pairing, encapsulation technologies, and targeted delivery systems‐remains an evolving field [37, 190, 191]. Maintaining microbial viability throughout processing, storage, and gastrointestinal transit remains a significant quality‐control challenge for probiotic products. Jin and Wang [192] highlighted low probiotic stability and viability as important limitations affecting the application of probiotics in food products, emphasizing the role of encapsulation and other stabilization strategies in improving survival. For prebiotics, product quality and regulatory standardization also remain important considerations, particularly because commercially marketed products may vary in composition, formulation, and substantiation of health claims [193].

### 6.7. Challenging the “Synergistic Superiority” Assumption for Synbiotics

A recurring assumption in the synbiotic literature is that combining probiotics and prebiotics can provide synergistic benefits beyond those achieved by either component alone. However, this assumption requires critical re‐examination. The ISAPP consensus distinguishes between complementary and synergistic synbiotics, with true synergy requiring evidence that the combined intervention provides greater effects than the individual components [190].

Smolinska et al. [194] reviewed 12 RCTs directly comparing synbiotics with the corresponding probiotic alone; only four (33%) reported statistically significant superiority of synbiotics, whereas five found no significant difference and three showed nonsignificant trends favoring synbiotics. Similarly, a 2024 network meta‐analysis of 68 RCTs in patients with type 2 diabetes found that synbiotics appeared more beneficial than probiotics or prebiotics for some outcomes; however, most effects were trivial or clinically unimportant, and the certainty of evidence was low to very low [195]. Evidence for synbiotic‐specific effects remains limited.

Fagnant et al. [196] identified only one RCT evaluating a synbiotic intervention for the prevention of gastrointestinal infections in nonelderly adults, limiting conclusions regarding its independent clinical benefit. Recent evidence also highlights substantial heterogeneity in synbiotic interventions, with variations in probiotic strains, prebiotic substrates, doses, and treatment durations that may contribute to inconsistent effects across studies [197].

Collectively, these findings suggest that combining a probiotic with a prebiotic does not consistently confer additional clinical benefits. Moreover, statistical significance does not necessarily indicate clinically meaningful superiority, particularly when outcomes are based on surrogate biomarkers or short‐term measures. Therefore, claims of synbiotic superiority should be evaluated in terms of both the magnitude and clinical relevance of observed effects.

## 7. Future Prospective

Extensive evidence supports the role of probiotics and prebiotics in maintaining gut homeostasis and systemic health. Despite the progress in understanding their mechanisms of action, several areas require exploration. First, considerable interindividual variability in gut microbiota composition, dietary habits, genetics, age, and environmental exposures influences treatment outcomes, emphasizing the need for personalized microbiome‐based interventions [198].

Second therapeutic efficacy is highly strain‐ and dose‐specific, making it inappropriate to generalize the benefits of one microorganism to another [199]. Optimization of synbiotic formulations should focus on effective probiotic‐prebiotic combinations, dosages, and delivery methods, whereas multistrain and multisubstrate synbiotics may offer broader gut modulation [200].

Mechanistic studies should focus on investigating systemic effects of SCFAs on distant organs beyond GIT [35]. Safety, regulatory, and technological considerations remain crucial, requiring long‐term evaluations and innovations to improve stability and delivery [201]. Future research should prioritize large, multicenter clinical trials with standardized protocols to identify optimal microbial strains, dosages, treatment durations, and patient populations [202]. The integration of multiomics technologies, including metagenomics, metabolomics, transcriptomics, and proteomics, with artificial intelligence and machine learning may facilitate the development of precision microbiome therapies tailored to individual patient characteristics [203]. Additionally, emerging approaches involving next‐generation probiotics, postbiotics, engineered microorganisms, and microbiome‐derived metabolites warrant further investigation, particularly to establish their efficacy, safety, and translational feasibility in well‐controlled human studies [204–206].

## 8. Conclusion

Overall, probiotics and prebiotics represent promising approaches for modulating gastrointestinal microbiota dysbiosis and may serve as complementary or, in selected clinical contexts, alternative strategies to conventional antibiotic therapy. However, their effects are not universally consistent and remain dependent on probiotic strain, prebiotic substrate, host characteristics, baseline microbiota, disease context, and intervention dose and duration. Although some applications are supported by clinical evidence, other proposed benefits remain primarily mechanistic or are supported by limited and heterogeneous clinical studies. Accordingly, findings from in vitro and animal models should not be assumed to translate directly into clinically meaningful outcomes and statistically significant changes in microbiota composition or biomarkers do not necessarily indicate therapeutic benefit. Future research should therefore prioritize well‐designed, adequately powered clinical trials that evaluate clinically meaningful outcomes, determine which patients are most likely to benefit from these interventions, and clarify strain‐ and context‐specific effects. Such evidence will be essential for defining where microbiota‐targeted interventions can provide reliable clinical benefit and where they should remain considered promising but insufficiently established approaches.

**Nomenclature**
AAD: antibiotic associated diarrhea
AD: Alzheimer′s disease
AhR: aryl hydrocarbon receptor
AMPs: antimicrobial peptides
AMR: antimicrobial resistance
CD: Crohn′s disease
DCs: dendritic cells
FDA: Food and Drug Administration
FOS: fructooligosaccharides
FXR: farnesoid X receptor
GALT: gut‐associated lymphoid tissue
GIT: gastrointestinal tract
GLP1: glucagon‐like peptide‐1
GOS: galactooligosaccharide
IBD: inflammatory bowel disease
IECs: intestinal epithelial cells
ISAPP: International Scientific Association for Probiotics and Prebiotics
LPS: lipopolysaccharides
MAMPs: microbe‐associated molecular patterns
MapA: Mucin adhesion‐promoting proteins
MLN: mesenteric lymph nodes
MUB: mucus‐binding proteins
NLRs: NOD‐like receptors
PD: Parkinson′s disease
pLAB: probiotic lactic acid bacteria
PRRs: pattern recognition receptors
PYY: peptide YY
SCFAs: short‐chain fatty acids
TGR5: Takeda G protein‐coupled receptor 5
TLRs: Toll‐like receptors

## Author Contributions

Tigistu Goa: conceptualization, literature review, writing—original draft preparation. Tesfaye Wolde and Elshaday Sahilu: review, editing, validation, and critical revision of the manuscript.

## Funding

No funding was received for this manuscript.

## Disclosure

All authors reviewed and approved the final version of the manuscript.

## Ethics Statement

The authors have nothing to report.

## Consent

The authors have nothing to report.

## Conflicts of Interest

The authors declare no conflicts of interest.

## Data Availability

All data and information supporting the findings of this review are included in the manuscript. Additional information is available from the corresponding author upon reasonable request.
